# Predicting beef diet nutritional composition and intake from rumen metagenomic profiles

**DOI:** 10.1016/j.aninu.2025.10.005

**Published:** 2026-01-06

**Authors:** Santiago N. Saez-Torillo, Rebecca Danielsson, Tuan Q. Nguyen, Joana Lima, Matthew A. Cleveland, Rainer Roehe, Marina Martínez-Álvaro

**Affiliations:** a Institute of Animal Science and Technology, Universitat Politècnica de València, Valencia 46022, Spain; b Swedish University of Agricultural Sciences, Uppsala 750 07, Sweden; c Scotland's Rural College, Edinburgh EH9 3JG, UK; d Genus Plc., DeForest 53532, WI, USA

**Keywords:** Feed composition, Microbiome composition, Prediction, Machine learning, Beef cattle

## Abstract

Knowledge of diet composition and intake levels in beef cattle is valuable for post hoc feed traceability and for more accurate modelling of the diet impact on methane emissions and performance traits. However, a direct measure of this information can be costly and labour-intensive and is not always feasible. In this study, rumen metagenomic data combined with machine learning algorithms were used to predict diet type, nutritional composition, and intake levels. An external validation to assess the generalizability of the models was also performed. Rumen samples were collected from 142 animals belonging to two breeds, Luing (*n* = 70) and Charolais crossbred (*n* = 72), with 425.6 ± 43.5 d old and 461.9 ± 70.2 kg body weight. The animals participated in a 56-d feeding trial and were assigned to diets differing in forage-to-concentrate ratio, with 72 animals receiving a concentrate-based diet and 70 receiving a forage-based diet. Liquid ruminal contents were collected immediately *postmortem* and subsequently subjected to metagenomic sequencing. Based on these sequences, the relative abundance of microbial genes (MGs), microbial genera (MTs), and phyla were determined. The log-ratio between the abundances of Verrucomicrobia and Chlorobi discriminated diet type with an average classification accuracy of 0.86 ± 0.05, while using the log-ratio transformed abundances of 4769 MTs and MGs as predictors reached 0.90 ± 0.05. All this microbiome information was used in a random forest model to predict continuous values for nutritional diet components starch, crude protein, neutral and acid detergent fibre, and metabolizable and gross energy with external validation prediction accuracy values between 0.77 and 0.83. Microbiome features important for prediction of diet components such as fibre and starch included *Mitsuokella*, *Selenomonas*, and MGs involved in flagellar assembly and aminoacyl-tRNA biosynthesis. Microbiome data were more informative for predicting the feed composition than the amount of feed consumed, which reached a prediction accuracy of 0.27 ± 0.12 for dry matter intake (DMI). However, microbiome data can still be used as a screening tool to classify DMI into low, medium, or high with a classification accuracy of 0.74. Incorporating dietary information into linear phenotypic and genetic models to predict methane production (MP) and DMI reduced root mean square error (RMSE) by 26.9% and 9.6%, respectively, in the phenotypic model. In the genetic model, only MP showed a reduction in RMSE, with a 31% improvement. These findings highlight rumen microbiome data as a valuable tool for the post hoc prediction of feed composition in beef cattle.

## 1 Introduction

The diet composition of beef cattle, together with their feed intake, plays a fundamental role in determining their performance, health, and overall productivity. Nutritional requirements, including energy, protein, fibre, and minerals, vary significantly across different physiological stages, such as growth, breeding, gestation, and lactation (Cabezas-Garcia et al., 2021; NRC, 2000). Moreover, diet influences the nutritional quality of animal-derived products; for example, meat and dairy products from forage-fed animals contain higher levels of beneficial unsaturated fatty acids, vitamins, and antioxidants (Alfaia et al., 2009; Aurousseau et al., 2004; Butler et al., 2011). As consumer demand for sustainably produced and traceable food increases, ensuring that animals have been fed according to certified standards becomes critical. Precise knowledge of diet composition of feed provided to an animal is important not only for understanding its growth, health status, and greenhouse gas emissions but also for ensuring traceability and market integrity of beef products, especially important for consumers willing to pay premium prices for quality products. Accurate knowledge of dietary intake information might be also useful for more accurate modelling of environmental effects on phenotypic traits and reliable phenotypic predictions and genetic evaluations (Silva-Neto et al., 2024) on diet-sensitive traits such as methane emissions. However, achieving this level of precision requires detailed tracking of individual feed intake and diet composition across large populations, something that remains challenging under commercial conditions.

Traditional assessment of the nutritional composition of animal diets is based on labour-intensive and time-consuming laboratory-based chemical and enzymatic methods. For example, fibre fractions are measured gravimetrically following sequential detergent and acid extraction steps; or energy fractions are measured by bomb calorimetry (Noblet et al., 2022), and in vivo digestibility trials. As an alternative, near-infrared reflectance spectroscopy (NIRS) methods are a valid and rapid, non-destructive tool for estimating diet components with high accuracy (De Boever et al., 1995), although both traditional and NIRS analyses require well-preserved, representative feed samples, and sampling bias becomes a major concern when dealing with heterogeneous diets. Precise intake assessment demands specialized infrastructure, such as automated feeding systems equipped with radio frequency identification technology, despite their precision, which are both expensive and logistically challenging (Brown-Brandl and Eigenberg, 2011). While accurate, these methods are impractical for large-scale monitoring and emphasize the need for proxies to estimate diet composition and intake more efficiently.

Diet is one of the most influential factors shaping the rumen microbiome composition in cattle (Henderson et al., 2015), followed by host genetics. The availability of specific nutrients directly modulates microbial community structure and fermentation patterns (Petri et al., 2013; Russell and Rychlik, 2001). Forage-based diets are primarily degraded by cellulolytic and fibrolytic bacteria like *Fibrobacter* and *Ruminococcus*, which break down cellulose and hemicellulose (Fernando et al., 2010; Miller et al., 2023; Ramos et al., 2021). In contrast, concentrate-based diets, which are rich in starch, favour the proliferation of amylolytic bacteria like *Prevotella*, *Butyrivibrio*, and *Selenomonas* (Fernando et al., 2010; Zebeli and Metzler-Zebeli, 2012). While numerous studies have compared the rumen microbiome of animals fed different commercial diets, the possibility of predicting the detailed nutritional composition of these diets (protein, fibre, or starch content) through externally validated algorithms remains underexplored. Rumen microbiome profiles could serve as a powerful tool for post hoc dietary monitoring in the era of precision livestock farming. However, despite the strong and well-documented links between dietary composition and ruminal microbial structure, the use of microbiome-based (and externally validated) predictive models to infer quantitative nutritional parameters remains underexplored area.

Microbial abundance can be quantified through several techniques. One of the main advantages of whole metagenome sequencing is that it offers access to both the taxonomic composition and the functional gene content of the microbial community (Durazzi et al., 2021), unlike metataxonomic approaches based on amplicon (16S rRNA) sequencing, which only provide taxonomic profiles. This dual insight enhances understanding of the functional potential of the rumen microbiome and its interactions with the host. Although sequencing-based approaches may still be relatively costly, the information they provide has numerous valuable applications. Beyond its potential to capture the diet consumed, rumen metagenomic data has proven useful to the early prediction of digestive disorders in cattle (Gebreyesus et al., 2020), productive performance (Malmuthuge and Guan, 2017) or the improved prediction of breeding values for methane emissions or average daily gain in beef (Martínez-Álvaro et al., 2022; 2024).

Ruminal metagenomic data includes a high number of variables with complex relationships. To handle this complexity, machine learning (ML) algorithms are increasingly used in microbiome research due to their ability to model non-linear and high-dimensional relationships between variables. Random forest (RF) is a widely used ensemble method that builds multiple decision trees, offering strong predictive performance even with noisy or sparse data (Breiman, 2001). These algorithms are particularly well-suited to microbiome data due to their flexibility and capacity to account for interactions and colinearity. However, ML algorithms are prone to overfitting and without rigorous external validation, model performance may be overestimated and its robustness compromised when applied to independent datasets or different production systems. Previous studies have applied ML models to predict host traits based on microbiome data; for example, Liu et al. (2022) reported accuracies ranging from 0.77 to 0.96 in their training dataset, when explaining fatty acid profiles. Similarly, the authors used RF to identify core microbial taxa (identified based on amplicon sequencing) that could predict methane emissions, feed efficiency, milk production or diet composition with *R*^2^ from 0.0 to 0.8 depending on the farm of origin. These results raise concerns about the consistence of the models across different environmental conditions. Validating these models across different conditions (e.g. different seasons or breeds) using independent external datasets is an essential step before their generalised implementation.

The aim of this study was to investigate the potential of rumen microbiome composition (at both taxonomical and functional levels) to predict dry matter intake (DMI) and diet composition: starch, crude protein (CP), neutral detergent fibre (NDF), acid detergent fibre (ADF), metabolizable energy (ME), and gross energy (GE); across different breeds and years in beef cattle. The predictive power of logistic regression and RF models is assessed, and external validation is conducted to evaluate their generalizability. Additionally, the integration of detailed dietary information into classical linear predictive models of economically and environmentally relevant traits in beef cattle (growth, intake, feed efficiency, and methane emissions) is explored to assess whether it improves prediction accuracy, or prediction ability in the case of genetic models.

## 2 Materials and methods

### 2.1 Animal ethics statement

The animal experiments were carried out at the Beef and Sheep Research Centre of Scotland's Rural College (SRUC) in Edinburgh, UK. These studies were approved by the SRUC Animal Experiment Committee and conducted in accordance with the requirements of the UK Animals (Scientific Procedures) Act, 1986.

### 2.2 Animals, diets, and performance traits records

The data were derived from two previous experimental trials conducted in 2012 and 2013, as reported by Duthie et al. (2018), Duthie et al. (2016), and Troy et al. (2015). These experiments followed a 2 × 2 factorial design, with two breeds, purebred Luing (LU) and crossbred Charolais (CH), and two diet types: with different forage to concentrate ratio of 500:500 or 80:920 in following referred to as mixed and concentrate diet, respectively. The distribution of the 142 beef cattle across diet and breed is presented in Table 1.

**Table 1:** Animal distribution by breed, year of trial, and diet and its differences.

Items	Year				Conc–Mix^2^	The percentage difference of mean^3^, %	Difference in units of SD^4^	SEM	*P*-value^5^
	2012		2013						
	Diet^1^		Diet^1^						
	Conc (36)	Mix (36)	Conc (36)	Mix (34)					
**Breed**^6^									
Charolais-X	18	17	19	17					
Luing	18	19	17	17					
**Diet component/intake**^7^**, g/kg DM**									
DMI, kg/d	10.9	9.7	10.0	10.7	0.25	2.4	0.18	0.24	0.290
Starch	414.4	278.7	440.1	284.1	145.9	41.2	7.73	3.19	<0.001
NDF	246.1	336.7	216.6	308.6	−91.7	33.1	5.50	2.80	<0.001
ADF	113.9	203.3	123.3	195.0	−80.7	50.8	9.78	1.39	<0.001
CP	133.8	138.6	124.9	134.9	−7.5	5.6	0.88	1.45	<0.001
ME, MJ/kg DM	12.7	12.0	12.1	11.3	0.74	6.1	1.84	0.067	<0.001
GE, MJ/kg DM	18.6	19.1	18.2	18.3	−0.29	1.6	0.67	0.074	<0.001

DM = dry matter; ADF = acid detergent fibre; CP = crude protein; GE = gross energy; ME = metabolizable energy; NDF = neutral detergent fibre; DMI = dry matter intake; SD = standard deviation; SEM = standard error of the mean.

1 Conc, forage:concentrate = 80:920; mix, forage:concentrate = 500:500.

2 Mean difference between concentrate and forage diets.

3 Percentage of the mean value represented by the difference between concentrate and forage diets.

4 Difference (conc−mix) divided by the SD, of the corresponding diet component.

5 *P*-value from a Welch *t*-test of the difference between diets.

6 Charolais-X, Charolais crossbreed; Luing, Luing breed.

7 Mean values are presented by diet and study year (2012 and 2013), together with the differences between diets. All ingredients were measured 4 weeks prior to slaughter; *n* = 142.

Although the experiment was carried out over two years, both were done in two phases. First, a performance test was carried out during their growing-finishing period, followed by the measurement of their methane emissions using six respiration chambers. Animals started the trial at an average body weight (BW) of 461.9 ± 70.2 kg and an average age of 425.6 ± 43.5 d. Prior to the trial, all animals were initially fed a mixed-based diet over a 5-week adaptation period and subsequently transitioned to the experimental diets. During this time, the animals were also acclimated to group housing conditions and trained to use the electronic feeding systems (HOKO, Insentec B.V., Marknesse, The Netherlands). The performance test phase consisted of 56 d feeding trial during which animals were fed ad-libitum one of the two diets. Animals maintained these experimental diets until slaughter. During the trial, feed intake was measured using the electronic feeding systems and after determination of the dietary dry matter expressed as kg/d for 56 d (DMI_56_). The BW of each animal was recorded weekly. Average daily gain for 56 d (ADG_56_) and mid-test BW were estimated fitting a linear regression of BW on test week. Feed conversion ratio for 56 d (FCR_56_) was determined as the ratio of average DMI_56_ (kg/d) to ADG_56_. Residual feed intake (RFI_56_) was calculated as the difference between observed DMI_56_ (kg/d) and ${\hat{\text{DMI}}}_{56}$ predicted by a linear regression model including ADG_56_, and mid-test metabolic BW (MBW; calculated as BW^0.75^) and subcutaneous fat depth at the 12th/13th rib as covariates. Methane production (MP, g/d) was measured after 56-d experimental period, using respiration chambers where the animals were allocated based on a randomized block design. Each steer was assigned to one of six respiration chambers for a period of 3 d, during which methane emissions were continuously monitored. One week before entering the respiration chambers, the animals were housed individually in training pens, identical in size and shape to the pens inside the chambers, to allow them to adapt to being housed individually. Throughout the 3-d measurement period, steers were fed once daily and provided ad libitum access to the same diet they consumed during the feeding trial. The average weight and age of the animals short after leaving the chamber were 653.5 ± 63.7 kg and of 529.2 ± 46.5 d, respectively. The methodologies employed adhered to established protocols, as outlined by Duthie et al. (2016, 2017) and Troy et al. (2015).

Second, to ensure that feed composition was determined in close alignment with the rumen sampling date, the diet composition of the feed and the feed intake recorded 4 weeks prior to slaughter were used (referred to as DMI to differentiate it from DMI_56_). The experimental diets were formulated using several ingredients, each of these was sampled separately to assess its chemical and energetic composition. Twice weekly, duplicate subsamples (approximately 350 g of fresh weight) of each feed ingredient (grass silage, whole crop barley, barley, wet distillers' grains, barley straw, molasses, and a mineral supplement) were collected. At each sampling point, a portion of the sample was analysed for dry matter (DM) in a forced-air oven. Another portion was frozen at −20 °C for subsequent chemical and calorimetric analyses. At the end of the trial, frozen samples from each ingredient were pooled and analysed for DM, CP, ADF, NDF, acid-hydrolysed ether extract (AHEE), and starch following standard methods (Ministry of Agriculture Fisheries and Food, 1992). The analysis of GE was done using a bomb calorimeter (Parr adiabatic bomb calorimeter, model 6200, Parr Instruments, Moline, IL, USA) using benzoic acid as a calibration standard. ME values were estimated using NIRS for silages, from net concentration of gross digestible energy and AHEE for distillers’ grains, or from tabulated values for minor ingredients such as straw and molasses (Thomas, 2004). The full list of diet ingredients and their compositional analyses is provided in Duthie et al., 2018, Duthie et al., 2016 and Troy et al. (2015). Twice weekly, the feed composition for each animal was calculated by multiplying the composition of the ingredients by the formulation of their diet. The final feed composition for each animal was obtained by averaging the 8 measurements corresponding to their 4 weeks before slaughter (Table 1).

### 2.3 Rumen sample collections and sequencing

At the end of the finishing period, animals were slaughtered in a commercial abattoir after 3 h of fasting. Post-mortem rumen digesta samples (approximately 50 mL of rumen fluid) were collected from each animal 30 min after slaughter by opening and draining the rumen, following procedures described by Auffret et al. (2017). Strained ruminal fluid (5 mL) were mixed with 10 mL phosphate buffered saline (PBS) containing glycerol (87%) and stored at −20 °C, following the protocol outlined by Rooke et al. (2014).

Microbial DNA was extracted from the rumen digesta samples as described in Yu and Morrison (2004), based on repeated bead beating with column filtration. DNA concentration and integrity were assessed using a NanoDrop ND-1000 spectrophotometer (ND-1000, Thermo Fisher Scientific Inc., Wilmington, DE, USA). Genomic libraries were sequenced on an Illumina HiSeq 4000 system, (HiSeq 4000, Illumina Inc., San Diego, CA, USA), at Edinburgh Genomics (Edinburgh, UK), generating paired-end reads (2 × 150 bp). The raw sequencing data are publicly available through the European Nucleotide Archive (www.ebi.ac.uk) under accession numbers PRJEB10338 and PRJEB31266 for the 2012 and 2013 experiments, respectively.

### 2.4 Bioinformatic analysis of rumen samples

The bioinformatic analysis of the sequencing data from 142 samples to obtain microbial genes (MGs) and microbial taxa abundances was conducted as described in Martínez-Álvaro et al., 2022a, Martínez-Álvaro et al., 2022b. In summary, to measure the abundance of known functional MG, whole-metagenome sequencing reads were quality-trimmed using Fastp (Chen et al., 2018) and assembled with MEGAHIT (Li et al., 2015). Protein-coding sequences were predicted using Prodigal (Hyatt et al., 2010), filtered to exclude incomplete proteins, and annotated against the Kyoto Encyclopedia of Genes and Genomes (KEGG) database (version 2020–10-04) (Kanehisa and Goto, 1999) using KofamScan (Aramaki et al., 2020). Reads were mapped to their respective assemblies using BWA-MEM (Li, 2013), and KEGG Orthology (KO) abundances were calculated with BamDeal (Barnett et al., 2011) and BEDTools (Quinlan and Hall, 2010). A total of 7976 KO abundances were identified.

For taxonomic annotation of the rumen samples, sequencing reads were aligned to a database containing cultured genomes from the Hungate 1000 collection (Seshadri et al., 2018) and RefSeq genomes (Pruitt et al., 2007) using Kraken (Wood and Salzberg, 2014) with default parameters. Forty-four microbial phyla and 1178 microbial genera (MTs), were identified and quantified. Counts of MGs, MTs, and microbial phyla were standardized as relative abundances. To focus on core microbiome functions and microorganisms, only MGs, MTs, microbial phyla present in at least 70% of the samples with a mean relative abundance > 0.001% were included. As a result of this filter, 3632 MGs, 1137 MTs, and 43 phyla were retained, accounting for 99.55% and 99.99% of the total identified counts, respectively. Zero relative abundances, accounting for 9.14% of the positions in entire MGs database, 3.83% of the MTs database, and 2.64% of the microbial phyla database, were imputed based on a bayesian-multiplicative replacement method by using zCompositions R package (Palarea-Albaladejo and Martín-Fernández, 2015). The relative abundance of MGs was then additive natural log-ratio transformed (3631 alr-MGs, Greenacre, 2018), using the ribulose phosphate 3-epimerase (*rpe*) gene as denominator. The Procrustes correlation between the exact log-ratio geometry and the alr-transformed MGs approximation was 0.998. MT relative abundances were centred log-ratio transformed (1137 clr-MTs, Greenacre, 2018), as no MT was found that, when used as a denominator in an alr transformation, preserved distances between samples (Greenacre et al., 2021). Specifically, the maximum Procrustes correlation between the exact log-ratio geometry and the approximate geometry generated by the alr-transformed MTs was ≤0.96.

### 2.5 Animal genotyping

For host DNA analysis, 6 to 10 mL of blood was collected from either the jugular or coccygeal vein on live animals (2012) or during slaughter in a commercial abattoir (2013). Blood was stored in tubes containing 1.8 mg ethylene diamine tetraacetic acid (EDTA) per mL blood and immediately frozen at −20 °C. Genomic DNA was isolated from blood samples using the QIAsymphony DSP DNA Midi Kit (937255, QIAGEN GmbH, Hilden, Germany) according to the manufacturer's instructions. Quality of the DNA samples was checked using a NanoDrop ND-1000 spectrophotometer (ND-1000, Thermo Fisher Scientific Inc., Wilmington, DE, USA). Genotyping was performed by Neogen Genomics (Ayr, Scotland, UK) using the GeneSeek Genomic Profiler (GGP) Bovine single nucleotide polymorphism (SNP) 50k Chip (GeneSeek, Lincoln, NE, USA). Genotypes were filtered for quality control purposes using PLINK version 1.09b (Purcell et al., 2007). SNPs were removed from further analysis if they met any of these criteria: unknown chromosomal location according to Illumina's maps (Matukumalli et al., 2009), SNP call rate lower than 95%, deviation from Hardy-Weinberg proportions (χ^2^ test *P*-value > 1 × 10^−8^), or minor allele frequency lower than 0.05. No animals were removed for showing a call rate lower than 90%. Missing SNP genotypes were imputed using the Beagle 5.2 software (Browning et al., 2018). After imputation and filtering, 38,807 SNPs remained for the analyses.

### 2.6 Algorithm development and validation

#### 2.6.1 Algorithms to classify samples by diet type using microbiome data

With the aim of identifying a simple microbial biomarker based on microbial phyla abundances in the rumen to determine the diet type (mixed or concentrate). A logistic regression was fitted using diet type as the response variable and a single log-ratio between the abundances of two microbial phyla as the predictor. The best log-ratio between two microbial phyla was selected using the Selbal function from the R package Selbal (Rivera-Pinto et al., 2018). Selbal is a stepwise logistic regression algorithm programmed to identify the best two groups of variables, which combined in a single log-ratio best associates to a given response variable. To maintain simplicity and interpretability, the maximum size of each group was set to 1, that is, the abundance of one microbial phylum in the numerator and one in the denominator. To evaluate the classification accuracy of the model, a validation approach was employed, dividing the dataset into training (70%) and testing (30%) sets balanced by diet. The model was fitted using the training set and validated on the external test set to calculate the model's classification accuracy and the area under the curve (AUC). Classification accuracy was determined as the proportion of right classifications in the testing set. The fitting and validation process was repeated 100 times (using 100 different training and testing sets) to calculate the mean and standard deviation (SD) of the AUC and classification accuracy across the 100 external testing sets. In each iteration, the two selected phyla were also recorded.

In addition, a RF algorithm (R package random forest, Liaw and Wiener, 2002) was applied using diet type as the response variable and 1137 clr-MTs and 3631 alr-MGs as predictors. To keep consistency, in this analysis, the training and test datasets were the same as in the previous analysis in which the predictor was log-ratio between two phyla. The training set was used to fit the RF model and tune the hyperparameters *mtry* (values tested: 69, 238, 477, and 794), *ntree* (fixed at 500), *nodesize* (values tested: 2, 4, 6, 8, and 10), and *maxnodes* (values tested: 5 and 10). Cross-validation was performed for the hyperparameter optimization process within the training and five rounds of optimization. The set of hyperparameters that achieved the highest average AUC across the rounds was selected. Once the optimal hyperparameters were identified, they were used to train models over 100 iterations. In each iteration, different training and testing sets were used (as done in the diet determination analysis), allowing for the calculation of the mean and SD of the AUC and classification accuracy across multiple simulated external test sets. The importance of each variable in each of the 100-models fitted was extracted using a permutation approach provided by the random forest package. In this approach the out-of-bag error is compared before and after permuting the values of that variable. From the importance values obtained across 100 iterations, variables assigned an importance greater than zero in at least 70% of the fitted models were identified. For these variables, the mean importance was calculated, excluding any zero, and the variables were ranked accordingly. To facilitate the interpretation of the most important MGs differentiating between diets (top 100 in the mean importance ranking), their pathways were identified using the R package clusterProfiler (Yu et al., 2012).

#### 2.6.2 Algorithms to predict the diet composition and intake using microbiome data

##### 2.6.2.1 Continuous prediction

The ability of the microbiome to predict animals’ DMI (kg/d) and the concentration of diet components in the consumed feed such as ADF (g/kg DM), CP (g/kg DM), ME (MJ/kg DM), GE (MJ/kg DM), NDF (g/kg DM), and starch (g/kg DM) was evaluated. To achieve this aim, each diet component was included as the response, and the 1137 clr-MTs, 3631 alr-MGs, and the diet type predicted by the previous RF classification model were included as predictors in an RF model. The model fitting and validation procedure was the same as that used for diet classification, this time selecting the hyperparameter set that achieved the lowest mean RMSE across the 5 cross-validation rounds. Once the optimal hyperparameters were set, models were trained and tested over 100 iterations to compute the mean and SD of RMSE and prediction accuracy across the 100 testing sets. As with diet discrimination, the most important variables for predicting the diet components and the DMI were identified based on their permutation importance. Variables identified as important for prediction of different diet components were explored in a Venn Diagram using ggVennDiagram R package.

##### 2.6.2.2 Categorical prediction

The ability of RF to classify animals based on their DMI and concentration of their diet components were evaluated. For this analysis, each animal observation was categorised according to each variable into high, medium and low, depending on their values being in the top, medium or lower third of the variable's range (Table 2).

**Table 2:** Distribution number of animals across categories.

Items^1^	Categories^1^		
	Low	Medium	High
ADF	72		70
CP	13	18	111
GE	23	70	49
ME	34	59	49
NDF	56	16	70
Starch	70		72
DMI	39	81	22

ADF = acid detergent fibre; CP = crude protein; GE = gross energy; ME = metabolizable energy; NDF = neutral detergent fibre; DMI = dry matter intake.

1 Low, first tercile of the range of concentration of each component or intake; medium, second tercile; high, third tercile; *n* = 142.

Then, RF model was used to predict the categorical variables, including as predictors the 4767 alr-MGs and clr-MTs as well as the diet type predicted by the previous RF classification model. The hyperparameters were selected based on a weighted precision metric, where misclassification based on a cost-sensitive learning (Ling and Sheng, 2010), i.e., penalisations for misclassifications between categories low and high were twice as heavy as those between adjacent categories (low to medium or medium to high). The process of optimization and validation of the models was done as in the diet type prediction.

### 2.7 Evaluating the benefit of including dietary information into phenotypic and genetic models for performance traits

To assess the importance of accounting for the diet composition consumed by the animals in predicting performance traits: ADG_56_, FCR_56_, RFI_56_ and DMI; and one environmental trait: MP, two predictive approaches were conducted. The first approach was a phenotypic prediction using a linear model including breed and trial as fixed effects (*glm* function from R stats package). The second approach was a genomic prediction, using BLUP model. The model included breed and year as fixed effects and additionally incorporated a random animal genomic effect. This analysis was performed using the gibbsf90 software (Misztal et al., 2002).

To identify the most adequate dietary information (i.e., diet components and diet type) to be included in the model of each trait, stepwise regression was performed using the stats package in R (Bolar, 2019). As a result, only the most relevant diet information (diet type as a fixed effect and diet component concentrations as covariates) were retained for each trait. In both approaches, the models were fitted with and without the dietary information using a training set (70% of the data), and they were compared based on their root mean square error (RMSE) and prediction accuracy. The prediction ability of the genetic model was calculated as the correlation between the estimated breeding values (EBVs) and the EBVs plus the error of the model. The database was randomly split 100 times into training and testing sets. Each model underwent multiple external validations using 100 training and 100 testing sets to calculate performance metrics.

## 3 Results

### 3.1 Discrimination between animals fed with mixed or concentrate-based diets using the microbiome

**Fig. 1:**
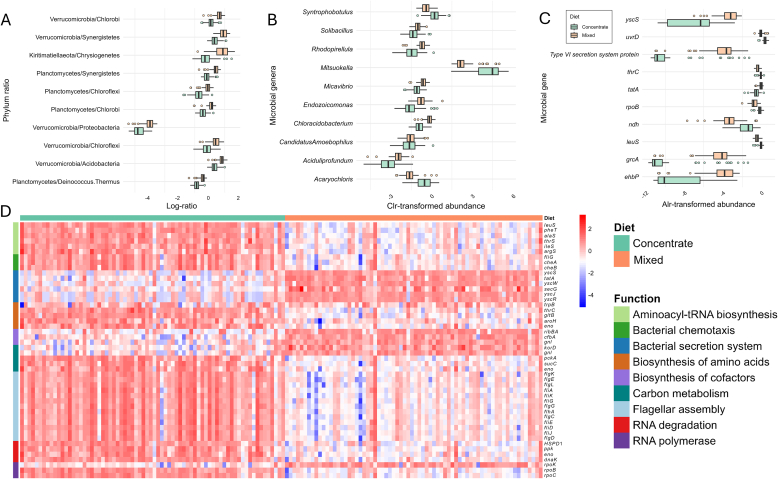
Diet discrimination and important variables (*n* = 142). (A) Distribution of the different log-ratios between two phyla selected by the Selbal stepwise forward selection algorithm as the best discriminant of diet across 100 iterations. (B) Centered log-ratio (clr) transformed abundance of the 10 most important microbial genera for diet discrimination based on random forest. (C) Additive log-ratio (alr) transformed abundance of the 10 most discriminative microbial genes (MGs) between diets based on random forest. (D) Heatmap of additive log-ratio (alr) transformed abundance of the most important MGs for the discrimination between diets with random forest, grouped by their metabolic pathway. The left axis shows the protein names encoded by these genes. Concentrate, forage:concentrate = 80:920; mixed, forage:concentrate = 500:500.

The rumen microbial composition of animals fed either a mixed-based or a concentrate-based diet showed differences between-phyla proportions (Fig. S1). Based on these differences, a microbial biomarker at the phylum level capable of discriminating between animals consuming mixed-versus concentrate-based diets was sought. Over 100 iterations, the log-ratio of abundances of Verrucomicrobia and Chlorobi was selected as the most effective discriminating biomarker in 67% of the cases. This ratio was higher in animals fed a concentrate-based diet compared to those on a mixed-based diet (0.50 ± 0.01 vs. 0.30 ± 0.01, *P* < 0.001, Fig. 1A). The Verrucomicrobia/Chlorobi log-ratio was able to classify diet type with an average AUC of 0.86 ± 0.05 and the same accuracy across multiple external test sets. The second most important discriminator was Verrucomicrobia/Synergistetes, which was chosen in only 9% of the iterations, while other ratios were selected at lower frequencies. For instance, the log-ratios Kiritimatiellaeota/Chrysiogenetes, Planctomycetes/Synergistetes, and Planctomycetes/Chloroflexi were selected 7%, 6%, and 5% of the partitions respectively. All identified log-ratio phyla, which were able to distinguish between the two diets are presented in Fig. 1A.

Surprisingly, using the whole microbiome dataset (3632 alr-MGs and 1137 clr-MTs) as predictor led accurate distinction of diet types, achieving marginally higher AUC and classification accuracy of 0.90 ± 0.05 across 100 external validations. The importance analysis identified 540 variables out of 4769, of which 107 correspond to clr-MTs and 433 to alr-MGs (Table S1). The MGs dominated the top of the ranking based on importances. Of the 434 alr-MGs identified as important for diet type discrimination (top 10 shown in Fig. 1C), the most important were *leuS*, type VI secretion system protein, *rpoB* and *yscS*, all with more alr-abundance in mixed-fed animals, except for *rpoB* which was more alr-abundant in concentrate. To facilitate the functional interpretation of the results, the most important MGs (top 100) were also grouped by metabolic pathways (Fig. 1D). Seventy-one out of 100 MGs were assigned to 67 enriched pathways, and 9 of these pathways were overrepresented (>3 important MGs involved in the pathway). Among them, the flagellar assembly pathway was notably overrepresented (13 MGs), and all the MGs were more abundant in animals fed concentrate-based diets, suggesting alterations in cell motility. All the MGs classified in aminoacyl-tRNA biosynthesis, RNA degradation, and bacterial chemotaxis pathways were also more abundant in the concentrate diet. In contrast, all the MGs in the bacterial secretion system and cofactor biosynthesis pathways were more abundant in the mixed diet. Additionally, MGs involved in carbon metabolism, amino acid biosynthesis and RNA polymerase pathways exhibited different abundance patterns in the two diet types.

The highest-ranked clr-MTs variable, *Mitsuokella*, appeared at 33rd place in the ranking followed by *Acaryochloris*, ranked 40th, both more clr-abundant in the rumen of animals fed a concentrate-based diet than those fed a mixed-based diet. Conversely, *Chloracidobacterium* (ranked 34th), followed closely by *Solibacillus* (38th), *Aciduliprofundum* (39th), and *Endozoicomonas* (47th), were consistently more abundant in animals receiving the mixed diet than in those receiving the concentrate diet.

### 3.2 Prediction of diet feed components consumed by animals based on the microbiome

The microbiome was able to predict all the feed components with high accuracy (Table 3). The prediction accuracies achieved in the testing sets ranged between 0.77 (ADF) and 0.83 (CP), with RMSE values of 27.14 g/kg DM for ADF, 5.89 g/kg DM for CP, 0.32 MJ/kg DM for GE, 0.34 MJ/kg DM for ME, 31.24 g/kg DM for NDF, and 46.54 g/kg DM for starch. Predictions were consistent across the 100 testing sets, with variations of approximately 0.07 in prediction accuracy. The amount of feed consumed (DMI) measured during 4 weeks prior to slaughter, was predicted by the microbiome with a prediction accuracy of 0.27 ± 0.12, and a RMSE of 1.35 kg/d across 100 external validation.

**Table 3:** Predictive performance for diet components and intake (DM basis)^1^.

Items^1^	Continuous prediction		Categorical prediction	
	Prediction accuracy	RMSE	Classification accuracy	AUC
**Diet components, g/kg**				
ADF	0.77 ± 0.08	27.14 ± 3.43	0.84 ± 0.05	0.95 ± 0.03
NDF	0.78 ± 0.07	31.24 ± 3.82	0.78 ± 0.06	0.85 ± 0.04
CP	0.83 ± 0.08	5.89 ± 0.86	0.88 ± 0.04	0.87 ± 0.05
Starch	0.80 ± 0.07	46.54 ± 5.58	0.82 ± 0.05	0.95 ± 0.03
GE, MJ/kg	0.80 ± 0.07	0.32 ± 0.03	0.77 ± 0.05	0.82 ± 0.05
ME, MJ/kg	0.81 ± 0.06	0.34 ± 0.03	0.83 ± 0.05	0.90 ± 0.03
DMI, kg/d	0.27 ± 0.12	1.35 ± 0.11	0.74 ± 0.04	0.70 ± 0.06

ADF = acid detergent fibre; CP = crude protein; GE = gross energy; ME = metabolizable energy; NDF = neutral detergent fibre; DMI = dry matter intake; RMSE = root mean squared error; AUC = area under the curve.

1 Mean (± standard deviation) prediction accuracy and RMSE, for continuous prediction, and classification accuracy and AUC, for categorical prediction, across 100 testing iterations in the 142 animals.

The RF model demonstrated good performance when diet components were classified into low, medium, or high levels, with a particularly strong improvement in the classification accuracy of DMI (Table 3). In this trait, the model achieved an AUC of 0.70 and a classification accuracy of 0.74 in external validation. The AUC scores for the diet components ranged from 0.82 (GE) to 0.95 (ADF and starch), while classification accuracy values varied between 0.77 (GE) and 0.88 (CP). As in their continuous counterparts, performance remained consistent across the 100 external validation iterations, with SD of up to 0.06. The most important microbiome prediction variables for classified diet components largely agreed with those selected based on their continuous values, and therefore, only the variables of the latter will be presented in this paper.

**Fig. 2:**
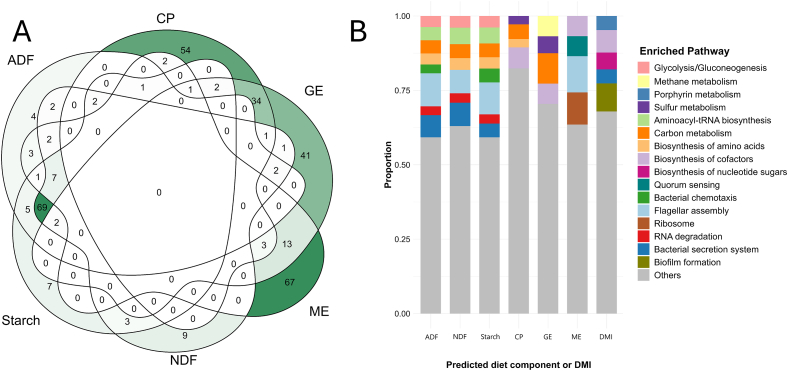
Prediction of diet components and important variables. (A) Venn diagram of the 100 most important variables of each component of the diet (*n* = 142). (B) Overrepresented pathways of the top 100 microbial genes (MGs) most relevant to each diet component and dry matter intake (DMI). Pathways with fewer than 4 MGs were grouped under “Others.” The number of MGs assigned to each diet component was as follows: 69 for ADF, 67 for STARCH, 63 for NDF, 51 for ME, 56 for GE, 62 for CP, and 56 for DMI. The figure represents the proportion of MGs associated with each pathway with respect to the total number of MGs assigned to each diet component. ADF = acid detergent fibre; CP = crude protein; GE = gross energy; ME = metabolizable energy; NDF = neutral detergent fibre.

The analysis of microbial variables relevant to predicting diet components showed that nearly all variables contributed to the models. Specifically, 4768 out of 4769 variables were relevant for ADF, NDF, CP, and GE, 4767 for ME, and 4748 for starch (Table S2). The test model included both microbiome information and the previously predicted diet type. The predicted diet type ranked among the top 25 most important variables for the prediction of ADF, NDF, and starch, which presented large differences between diets (33.1%–50.8% of the mean, exceeding 5 SD), but not in CP, ME, and GE, in which differences were smaller (1.6%–6.1% and below 2 SD) (Table 1). Among the top 100 microbial variables predicting each diet component, 338 unique variables were identified, including 252 MGs, 85 MTs, as well as the predicted diet. The components ADF, NDF, and starch shared 69 out of the 100 top important variables (Fig. 2A), whereas GE and CP shared 34 and GE and ME 13. Despite these common predictors, each diet component also presented unique microbial variables, with ME showing the highest number (67), followed by CP (54) and GE (41). Expanding the analysis to 200, 300, or 500 variables preserved similar proportions of shared variables among the diet components, demonstrating the robustness of these results across different variable selection thresholds. Some of the most important MTs ( Table S2) for prediction of starch, NDF, and ADF were *Mitsuokella*, *Endozoicomonas*, *Shingulisphaera*, *Micavibrio*, *Acaryachloris*, *Chloracidobacterium*, *Thermogymnomonas*, and also, the highly abundant *Fibrobacter* and *Selenomonas*. Important genera for prediction of CP and GE, were *Methanosphaerula*, *Synechocystis*, *Thermotoga*, *Blastococcus*, *Palaeococcus*, *Beijerinckia*, *Methylotenera*, and *Megasphaera*, the latter also important explaining ME. Finally, ME and GE shared important MTs as *Thermobifida*, *Chryseobacterium*, *Petrimonas*, *Clostridioides*, *Lactobacillus*, *Saccharomonospora*, *Ottowia*, *Elusimicrobium*, and *Sharpea.* From a functional perspective, Fig. 2B shows the overrepresented microbial pathway analysis based on most important MGs (top 100) for the prediction of each component. Not all MGs were assigned to pathways by the clusterProfiler package. The number of annotated MGs for each diet component was 67 for prediction of starch, 63 for NDF, 51 for ME, 56 for GE, 62 for CP, and 69 for ADF. Furthermore, 93 different pathways were identified in total but only 16 were overrepresented (>4 important MGs involved in the pathway). Seven microbial pathways were important in all diet components: amino acid biosynthesis, starch and sugar metabolism, quorum sensing, glycine, serine, and threonine metabolism, pyrimidine metabolism, nucleotide metabolism, and cofactor biosynthesis. As expected, the microbial pathways important for ADF, NDF and starch were very similar, and flagellar assembly was the most overrepresented pathway, followed by bacterial secretion system. Interestingly, flagellar assembly was also the most overrepresented pathway for ME. CP was overrepresented for MGs involved in biosynthesis of cofactors and carbon metabolism; and these pathways were also important for GE, together with methane metabolism pathway, including MGs as *fwdA*, *mtaA*, *mtaC*, *hdrB2*, *and hdrA2*. Both also share the top two MGs: *napD* and *dmsA*.

For DMI prediction, 3743 variables were identified as important, whereas the predicted diet type was not included, as animals fed with either concentrate or mixed had similar DMI on average (10.2 kg/d for the concentrate-based diet and 10.5 kg/d for the mixed-based diet). From a taxonomic perspective, *Syntrophus* and *Verticillium* were the two most important MTs to predict this trait; and from a functional perspective, MGs involved in biofilm formation were the most overrepresented (10 MGs), followed by biosynthesis of cofactors (8), biosynthesis of nucleotide sugars (6), and bacterial secretion system (5), among others (Fig. 2B).

### 3.3 Benefits of including dietary information in models fitting performance and emission traits

On average, animals exhibited an ADG_56_ of 1.54 ± 0.25 kg/d, an FCR_56_ of 7.45 ± 1.19 kg DM/kg BW gain, an MP of 178.96 ± 51.09 g/d, and an RFI_56_ centred around zero (0.00 ± 0.67 kg/d), based on records from 135 to 142 individuals depending on the trait. To evaluate the contribution of dietary information to the prediction of these traits, two types of linear models were tested. The first model included only fixed effects of year and breed (referred to as the basic model), while the second incorporated additional dietary information, diet type and diet components (referred to as the diet-informed model). Stepwise regression was performed using all available dietary information to identify the most relevant variables for each trait, and the final predictors retained in each model are summarized in Table 4. The predictive performance of both models was then assessed over 100 iterations of external validation, with results for training and testing datasets presented in Table 5.

**Table 4:** Predictors selected by stepwise regression to explain each phenotypic trait, together with fixed effects breed and year of trial.

Traits^1^	Dietary information^2^
ADG_56_	GE + starch
DMI	Diet + GE + Starch + CP + ADF
FCR_56_	ME + CP
MP	Diet + ADF + CP
RFI_56_	Diet + ADF + CP + NDF

ADF = acid detergent fibre; CP = crude protein; GE = gross energy; ME = metabolizable energy; NDF = neutral detergent fibre; DMI = dry matter intake; MP = methane production; FCR = feed conversion ratio; RFI = residual feed intake; ADG = average daily gain.

1 Traits were measured during a 56-d feeding trial during finishing or immediately after (MP), except DMI, measured 4 weeks prior to slaughter.

2 Available predictors were diet (fixed effect with two levels, mixed and concentrate), ADF, CP, GE, ME, and NDF, as covariates.

**Table 5:** Results of prediction metrics model and diet-informed model^1^.

Items	Traits^2^				
	ADG**_56_**, kg/kg weight	DMI, kg/d	FCR**_56_**, kg/d	MP, g CH_4_/d	RFI**_56_**, kg/d
**Linear model**					
Prediction accuracy	0.36 ± 0.10	0.11 ± 0.09	0.52 ± 0.08	0.16 ± 0.11	0.32 ± 0.11
RMSE	0.23 ± 0.02	1.46 ± 0.12	1.01 ± 0.12	51.37 ± 4.76	0.64 ± 0.07
**Diet informed linear model**					
Prediction accuracy	0.41 ± 0.04	0.43 ± 0.12	0.53 ± 0.09	0.69 ± 0.06	0.43 ± 0.11
RMSE	0.23 ± 0.02	1.32 ± 0.13	1.00 ± 0.10	37.56 ± 3.89	0.61 ± 0.05
**Linear genetic model**					
Prediction ability^3^	0.17 ± 0.13	0.33 ± 0.12	0.14 ± 0.12	0.11 ± 0.12	0.00 ± 0.14
RMSE	0.19 ± 0.13	1.14 ± 0.79	0.79 ± 0.65	43.01 ± 28.55	0.52 ± 0.40
**Diet informed linear genetic model**					
Prediction ability^3^	0.20 ± 0.12	0.33 ± 0.13	0.18 ± 0.11	0.26 ± 0.11	0.04 ± 0.12
RMSE	0.18 ± 0.13	1.04 ± 0.71	0.81 ± 0.59	29.77 ± 22.28	0.50 ± 0.36

ADG = average daily gain; MP = methane production; FCR = feed conversion ratio; RFI = residual feed intake; DMI = dry matter intake; RMSE = root mean squared error.

1 Model included prediction accuracy, RMSE, and prediction ability in genetic models, evaluated in the testing set across traits for basic and diet-informed linear models for the 142 animals (mean ± standard deviation). Diet-informed model additionally included dietary information detailed in Table 4.

2 Traits were measured during a 56-d performance test trial during the growing-finishing phase (ADG_56_, FCR_56_, and RFI_56_) or at the end of finishing (MP) or measured 4 weeks prior to slaughter (DMI).

3 Prediction ability is the correlation between the estimated breeding values (EBVs) and the EBVs, plus the residual of the model.

In the phenotypic models, the inclusion of dietary information improved predictive performance DMI and MP compared with the basic model (Table 5). The most substantial improvement was observed for MP, where prediction accuracy increased from 0.16 ± 0.11 to 0.69 ± 0.06 and RMSE decreased from 51.37 ± 4.76 to 37.56 ± 3.89 g CH_4_/d (26.9% of mean decrease). DMI also showed a clear enhancement, with prediction accuracy rising from 0.11 ± 0.09 to 0.43 ± 0.12 kg/d and RMSE dropping from 1.46 ± 0.12 to 1.32 ± 0.13 kg/d (9.58% of mean decrease). RFI displayed a tendence of improvement in prediction accuracy (from 0.32 ± 0.11 to 0.43 ± 0.11). No clear improvement was observed for ADG or FCR.

Including dietary information in the genetic linear models consistently reduced RMSE (Table 5). MP showed the largest improvement, with RMSE decreasing from 43.01 ± 28.55 g/d in the basic model to 29.77 ± 22.28 g/d in the diet-informed model, representing a 31% reduction. This was accompanied by an increase in the prediction ability of this trait, from 0.11 ± 0.12 to 0.26 ± 0.11. Similarly, DMI and RFI_56_ showed a tendency toward reduced RMSE in the diet-informed model (1.04 ± 0.71 and 0.50 ± 0.36 kg/d, respectively) compared to the basic model (1.14 ± 0.79 and 0.52 ± 0.40 kg/d), although without significant changes in their prediction ability. For FCR_56_ and ADG_56_, prediction ability and RMSE values were similar between models.

## 4 Discussion

### 4.1 The potential of using microbiome data as a PLF tool to predict the composition of the diet

The microbiome is known to undergo changes in response to diet, host genetics, and geography, although diet is considered the most influential factor (Henderson et al., 2015). Beyond this established knowledge, it is demonstrated that microbiome composition serves as a direct reflection of the actual diet composition consumed by the animal in the 30-d period preceding rumen sampling. When using microbiome information as a predictor, the ML models achieved high prediction accuracies (from 0.77 to 0.83) for concentration of fibre, starch, protein, and energy. The algorithms are robust to different breeds (Charolais and Luing) and different experimental years (2012 and 2013) as the 100 different external tests were unbalanced by breed and year and external validation accuracies were still consistent (standard errors ranging from 0.06 to 0.08). The performance of these equations is subjected to rumen samples collected at slaughter and to predicting diet composition consumed late in life; extrapolation to other time periods or sampling methods (e.g., stomach tubes) needs to be tested. The applicability of these models is greatest in scenarios where ruminal microbiome data are available, but the dietary information is unavailable or cannot be accurately assigned to individual animals within herd. Since recording individual feed intake and collecting feed samples and its analysis is labour-intensive and costly, inferring feed intake and diet composition from the rumen microbiome presents a valuable alternative. This is particularly relevant in extensive grazing systems, where accurately recording feed intake and diet composition is challenging. It is important to note that the algorithms were calibrated based on data from diets with forage-to-concentrate ratios of 500:500 and 80:920, typical of intensive systems. Extending their application to other feeding systems such as wild and tropical pastures, regional by-products or alternative feedstuff, will require recalibration and validation using data from these systems. Importantly, even when dietary information of the animals is available, the ability to infer diet composition from microbiome profiles post hoc ingesta opens new possibilities of traceability in livestock production. This approach could be used to detect inconsistencies between declared and actual feeding practices. It may also be useful in quality certification systems where independent verification of feeding practices is required but difficult to achieve.

At the current state of beef industry, both MP and DMI are critical traits from both environmental and economic perspectives. Beef cattle have a significant environmental footprint (FAO, 2022), and efforts to reduce their emissions are made from multiple animal science disciplines. Economically, feed represents the largest cost for commercial beef producers, accounting for approximately 60%–70% of variable production costs (Anderson et al., 2005; Baron et al., 2014). However, both MP and DMI are expensive traits to measure, as gold-standard methods (respiration chambers for MP and electronic feeders placed on scales for DMI) require important logistic and economic resources. As a result, these traits are often aimed to be predicted using alternative sources of information; and there is high research interest for studying how different factors influence these traits. This study demonstrates the importance of incorporating rigorous dietary information when modelling these traits. The results show that linear models for predicting DMI and MP perform better when dietary information is included in the model. For MP, the prediction accuracy increased from 0.16 to 0.69 when including diet type, ADF and CP in the model; and for DMI, it increased from 0.11 to 0.43 when including diet type, GE, starch, CP, and ADF. These improvements may vary with different initial predictor sets, which in this case was breed and year of experimental trial as fixed effects. As an example, Ellis et al. (2007), demonstrated that incorporating forage proportion, ADF and lignin into a CH_4_ prediction model which initially included DMI reduced root mean square percentage error from 25.6% to 14.4%. Dietary information can be also used for a more accurate modelling and correction of the environmental components in genetic evaluation models, decreasing the proportion of unexplained (or residual) variance. This is particularly important when diet and genetic group are confounding effects, i.e., animals from same herd fed with similar feed, or preferential dietary treatment to best animals. In these cases, acknowledging a detailed nutritional information could avoid bias in estimated breeding values. Another practical implication is the possibility of fitting genetic reaction norm models using the dietary composition as and environmental gradient, facilitating the consideration of genetic by environment interactions in regular evaluations. In this study, microbiome information is presented as a valuable proxy for obtaining dietary composition when it cannot be directly measured.

While collecting rumen samples entails practical challenges, sequencing cost is becoming increasingly accessible. Although the price of measuring microbiome data with whole metagenome-sequencing might result more costly than other approaches as 16S gene sequencing (108€ per sample vs. 44€, prices consulted June 10, 2025 on FISABIO), the functional information consisting on the abundance of 3631 MGs was key for the success of the diet component prediction, as more MGs helped to differentiate between diets or predict diet components than MTs, and also were more important and captured larger differential abundances between diets (Fig. 1). Thus, although relatively expensive, the large amount of information provided by whole metagenome sequencing of the microbiome, and its multiple applications, as early prediction of digestive disorders in cattle (Gebreyesus et al., 2020), productive performance (Malmuthuge and Guan, 2017), the improved prediction of breeding values of different traits (Martínez-Álvaro et al., 2022a, Martínez-Álvaro et al., 2022b, 2024) and now, the prediction of the diet environment–makes it cost–effective.

### 4.2 Intake prediction using microbiome data

An important result of this research is that microbiome composition is more sensitive to the type and composition of feed than to the amount of feed consumed by the individual animal. While prediction accuracies for diet components reached approximately 0.8, the accuracy for predicting DMI as a continuous trait was 0.27. However, using microbiome data to classify DMI into intake categories, low (e.g., <9.36 kg/d), medium (e.g., 9.36-11.58 kg/d), or high (e.g., >11.58 kg/d), improved the classification accuracy up of 0.70, suggesting that RF models built with microbiome data can still serve as a useful tool for broad screening of animals by intake level. The results align with findings from Delgado et al. (2019), who reported a prediction accuracy of 0.39 in an external test when predicting DMI as a continuous variable in dairy, based on a linear model incorporating microbiome-derived features. These results suggest that microbiome data alone may not be optimal for precise DMI prediction as a continuous variable, which may be influenced by additional factors beyond microbial profiles. Blake et al. (2023) proposed a RF model for DMI using animal-specific variables (average daily gain, age, weight, water intake, and sex) along with environmental parameters (temperature, radiation, and humidity), achieving *R*^2^ values ranging from 0.45 to 0.68 depending on the algorithm and in a single test set. Based on these findings, incorporating diet composition in this model could further improve the accuracy of the prediction, that if not available, can be accurately inferred from microbiome data.

### 4.3 The important microbial biomarkers that reflect the dietary information

Changes in microbial abundances due to differences in the diets were observed across multiple taxonomic and functional levels, including phyla, genera, and MGs. The two diet types in this study, with 500:500 and 80:920 forage-to-concentrate ratios differed in their NDF (231.4 vs. 322.4 g/kg), ADF (118.6 vs. 199.4 g/kg), and starch (427.0 vs. 281.4 g/kg) concentration, whilst had similar GE (18.4 vs. 18.7 MJ/kg), ME (12.4 vs. 11.7 MJ/kg), and CP (129.4 vs. 136.8 g/kg). Thus, microbial biomarkers important to discriminate between diets are, to a large extent, the same ones that are important for the linear prediction of the concentration of NDF, ADF, and starch, and will be discussed together. At phyla level, the analysis identified that a single log-ratio between Verrucomicrobia and Chlorobi abundances was an effective microbial biomarker to discriminate between mixed and concentrate-type diets with an AUC of 0.86 ± 0.05. Other discriminative log-ratios were also identified, with phylum Verrucomicrobia frequently included (e.g., Verrucomicrobia/Synergists or Verrucomicrobia/Proteobacteria). Verrucomicrobia phyla abundance is greater in animals fed with mixed compared to concentrate ([0.110 ± 0.038]% vs. [0.070 ± 0.024]%) and this has been observed in other studies (Pang et al., 2022, Wang et al., 2020). The MTs *Opitutus* and *Verrucomicrobium*, both within the phylum Verrucomicrobia, were identified as key MTs associated with diet differentiation and predictive of fibre and starch concentrations. Their clr-transformed abundances were higher in the mixed diet compared to the concentrate diet. Microorganisms classified within the phylum Verrucomicrobia possess a broad arsenal of carbohydrate-degrading enzymes, capable of breaking down complex polysaccharides present in mixed-based diets (Cardman et al., 2014; Rakitin et al., 2024; Sichert et al., 2020). As expected, other fibrolytic bacteria playing a crucial role in degrading lignocellulosic material like *Fibrobacter* genera (Fernando et al., 2010), *Chloracidobacterium* (Dadwal et al., 2019), and several fungi (e.g. *Alternaria* and *Trichosporon*) capable of degrading plant cell wall polysaccharides (Belanche et al., 2012) were more abundant in animals fed with mixed because of their association with the concentration NDF, ADF, and starch, which they also contributed to predict. From a functional perspective, several MGs encoding enzymes degrading complex carbohydrates were more abundant in mixed diets and largely contributed to the prediction of fibres and starch of the feed. This included glucose-6-phosphate 1-epimerase (identified as a CAZyme), a cellulase enzyme that breaks down cellulose into cellobiose or glucose by hydrolyzing β-1,4-glycosidic bonds, and *pepE*, which participates in the degradation of structural plant carbohydrates like pectin (Koike and Kobayashi, 2009). The rumen of animals fed with a diet high in NDF and ADF and low in starch contained a greater abundance of several MGs involved in the bacterial secretion system (*yscS*, *tatA*, *yscW*, *secG*, *yscJ*, and *yscR*). Specifically, these MGs encode key components of both Sec-dependent and Sec-independent translocation systems (Berks et al., 2000) and potentially, these systems can translocate active degradative enzymes (Artzi et al., 2017). In the mixed-fed group, also MGs *korD* (involved in oxidative decarboxylation reactions in anaerobic conditions) and *gnl* (encoding gluconolactonase that contributes to the conversion of complex sugars) were more alr-abundant encoding. These functional results reflect that microbial communities in mixed-fed cattle are adapted for the degradation and utilization of complex plant carbohydrates (Kanagasundaram and Scopes, 1992).

**Fig. 3:**
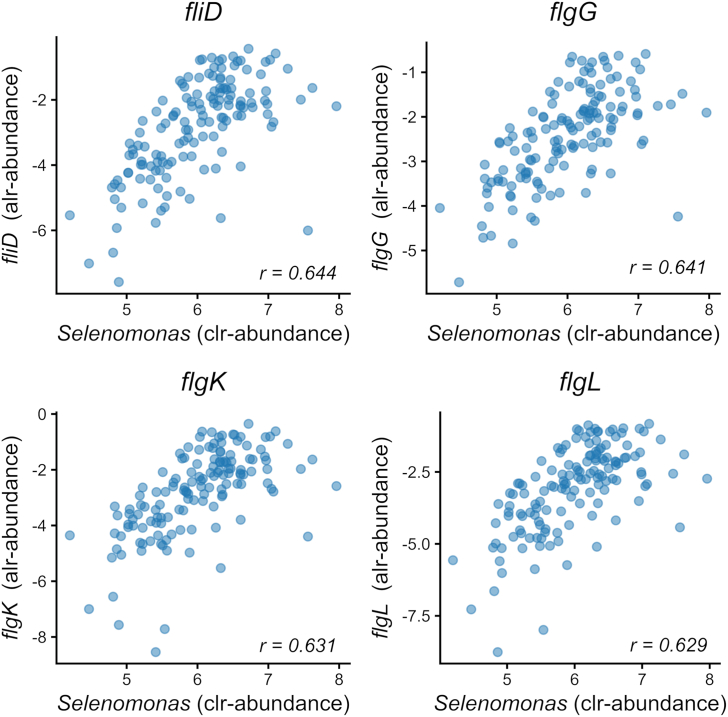
Flagellar assembly genes and their relationship with *Selenomonas*. Scatterplots showing the relationships between the centered log-ratio (clr)-transformed abundance of *Selenomonas* and the additive log-ratio (alr)-transformed abundances of 4 representative genes (*fliD*, *flgG*, *flgK*, and *flgL*) involved in the flagellar assembly pathway in 142 animals.The Pearson correlation coefficients (*r*) are indicated in each plot.

The rumen of animals fed with the concentrate diet, rich in starch and with lower concentration of ADF and NDF, showed greater clr-abundance of *Mitsuokella* (Fig. 1B). *Mitsuokella* was ranked also as top important in the prediction of NDF, ADF, and starch. This higher prevalence is expected, because is described that *Mitsuokella* produces phytase, an enzyme that breaks down phytate, a major component of cereal-based protein sources, particularly in concentrate diets (Tang et al., 2021). Amylolytic bacteria *Selenomonas*, a key starch- and sugar-fermenting genera that produces propionate (Fernando et al., 2010; Zebeli and Metzler-Zebeli, 2012) was also an important MT in the prediction of starch, ADF, and NDF; and more clr-abundant in the concentrate than mixed diet (6.41 vs. 5.56). Interestingly, in the functional analysis, 13 MGs associated with the flagellar assembly pathway were identified, all of which were more abundant in the rumen of cattle fed a concentrate-based diet. These MGs were also highly correlated with the clr-abundance of Selenomonas, with Pearson correlations ranging from 0.55 to 0.64 (Fig. 3), a genus known to comprise motile, flagellated bacteria (Bryant, 1956). MGs *leuS* involved in the aminoacyl-tRNA biosynthesis pathway was the most important variable in the 4 RF models (diets, ADF, NDF, and starch) and was also more abundant under concentrate feeding.

*Methylotenera*, a genus known for oxidising C1 compounds such as methanol and methylamines, was identified as a key MT associated with the prediction CP and GE in this study. This aligns with the detection of the *napD* gene, which encodes a chaperone essential for assembling NapA, the periplasmic nitrate reductase, enabling *Methylotenera* to utilise nitrate as a terminal electron acceptor (Mustakhimov et al., 2013). Furthermore, *Methylotenera* can assimilate methanol produced by other microbes, thereby competing with methanogenic archaea for this substrate (van Grinsven et al., 2021). At the same time, methanogenesis-related genes as important in GE and CP, such as *fwdA*, *mtaA*, *mtaC*, and *hdrB2* were identified.

*Fibrobacter* and *Chryseobacterium* were identified as important MTs associated for ME prediction, and they showed opposite correlations with ME *Fibrobacter* is negatively correlated (−0.51), while *Chryseobacterium* is positively correlated (0.51). This may be explained by *Fibrobacter*'s production of acetate, a less energy-efficient volatile fatty acid that promotes hydrogen release and methanogenesis (Ransom-Jones et al., 2012; Ungerfeld, 2020). In contrast, *Chryseobacterium*, though less studied in the rumen, produces fibrolytic enzymes (Tan et al., 2018) and may aid in secondary polysaccharide degradation, potentially enhancing energy availability through synergy with propionate-producing microbes.

## 5 Conclusion

This study highlights the potential of rumen metagenomic profiling as a powerful precision livestock tool for accurately reconstructing diet composition in beef cattle across breeds and experiments. By combining ML methods with metagenotypes, microbial and functional signatures were identified that not only discriminated between mixed- and concentrate-based diets with high precision (AUC up to 0.90 ± 0.05) but also predicted the concentrations of ADF, NDF, starch, CP, ME, and GE in the diet with high precision (accuracies of approximately 0.8). This information could be used for testing dietary intervention on individual animals, monitoring its effects, breeding and traceability. Functional profiles of the rumen were more often selected as important predictors in the model than its taxonomic counterparts. As a highlight, microbial genera (MTs) *Mitsuokella* and *Selenomonas* and 13 motility MGs correlated to the last genera, were associated to diet high in starch, and low in NDF and ADF. These results open new possibilities of traceability in livestock production, as can be used to detect inconsistencies between declared and actual feeding practices. The importance of including nutritional dietary information when fitting models to predict methane production and dry matter intake in beef was also highlighted, as it reduced the residual variance (RMSE) by 31% and 8.7%, respectively; when such information is not available, it can be predicted from microbiome data. Whilst microbiome data reflected the composition of the diet, it was less powerful for a continuous prediction of the amount of feed consumed (prediction accuracy 0.27). Still, it was a valid screening tool to classify animals in low, medium or high DMI, with accuracies of 0.74.

## CRediT authorship contribution statement

**Santiago N. Saez-Torillo:** Writing – review & editing, Writing – original draft, Visualization, Validation, Software, Formal analysis, Data curation. **Rebecca Danielsson:** Writing – review & editing, Methodology, Investigation. **Tuan Q. Nguyen:** Writing – review & editing. **Joana Lima:** Writing – review & editing. **Matthew A. Cleveland:** Writing – review & editing, Funding acquisition. **Rainer Roehe:** Writing – review & editing, Resources, Project administration, Methodology, Funding acquisition, Conceptualization. **Marina Martínez-Álvaro:** Writing – review & editing, Supervision, Software, Resources, Methodology, Funding acquisition, Conceptualization.

## Declaration of generative AI and AI-assisted technologies in the writing process

During the preparation of this work the author(s) used ChatGPT in order to improve readability and language of the work. After using this tool/service, the author(s) reviewed and edited the content as needed and take(s) full responsibility for the content of the publication.

## Declaration of competing interest

The authors declare the following financial interests/personal relationships which may be considered as potential competing interests: Matthew A. Cleveland is currently employed by Genus Plc., DeForest, Wisconsin 53532, USA.
