# Supplementary material for: Predicting beef diet nutritional composition and intake from rumen metagenomic profiles
Source: Anim Nutr. 2026 Jan 6;25:297–309. doi: 10.1016/j.aninu.2025.10.005 (PMC13081704; doi:10.1016/j.aninu.2025.10.005)
Supplement: Multimedia component 1 [file mmc1.docx]

**Table S3** Gene abbreviations and their corresponding full names.

| Abbreviations | Full names |
| --- | --- |
| *alaS* | Alanyl-tRNA synthetase |
| *argS* | Arginyl-tRNA synthetase |
| *aroH* | Chorismate mutase H |
| *cfbA* | Coenzyme F420 biosynthesis protein A |
| *cheA* | Chemotaxis histidine kinase A |
| *cheB* | Chemotaxis response regulator–methylesterase B |
| *dnaK* | Chaperone protein K |
| *eno* | Enolase |
| *flgC* | Flagellar basal-body rod protein C |
| *flgD* | Flagellar hook cap protein D |
| *flgE* | Flagellar hook protein E |
| *flgG* | Flagellar basal-body rod protein G |
| *flgK* | Flagellar hook-associated protein K |
| *flgL* | Flagellar hook-associated protein L |
| *flhA* | Flagellar biosynthesis protein A |
| *fliA* | Sigma-28 factor (flagellar sigma factor) |
| *fliD* | Flagellar filament cap protein D |
| *fliE* | Flagellar hook–basal body complex protein E |
| *fliG* | Flagellar motor switch protein G |
| *fliJ* | Flagellar export chaperone J |
| *fliK* | Flagellar hook-length control protein K |
| *gltB* | Glutamate synthase large subunit |
| *gnl* | Gluconolactonase |
| *HSPD1* | Heat shock protein 60 |
| *ileS* | Isoleucyl-tRNA synthetase |
| *leuS* | Leucyl-tRNA synthetase |
| *pheT* | Phenylalanine–tRNA synthetase beta subunit |
| *ppk* | Polyphosphate kinase |
| *rpoB* | RNA polymerase beta subunit |
| *rpoC* | RNA polymerase beta' subunit |
| *rpoK* | Alternative sigma factor K |
| *ribBA* | GTP cyclohydrolase II/riboflavin biosynthesis protein |
| *secG* | Protein translocase subunit G |
| *tatA* | Twin-arginine translocation protein A |
| *thrC* | Threonine synthase |
| *thrS* | Threonyl-tRNA synthetase |
| *trpB* | Tryptophan synthase beta chain |
| *yscJ* | Type III secretion system ring protein J |
| *yscR* | Type III secretion system protein R |
| *yscS* | Type III secretion system protein S |
| *yscW* | Type III secretion system protein W |


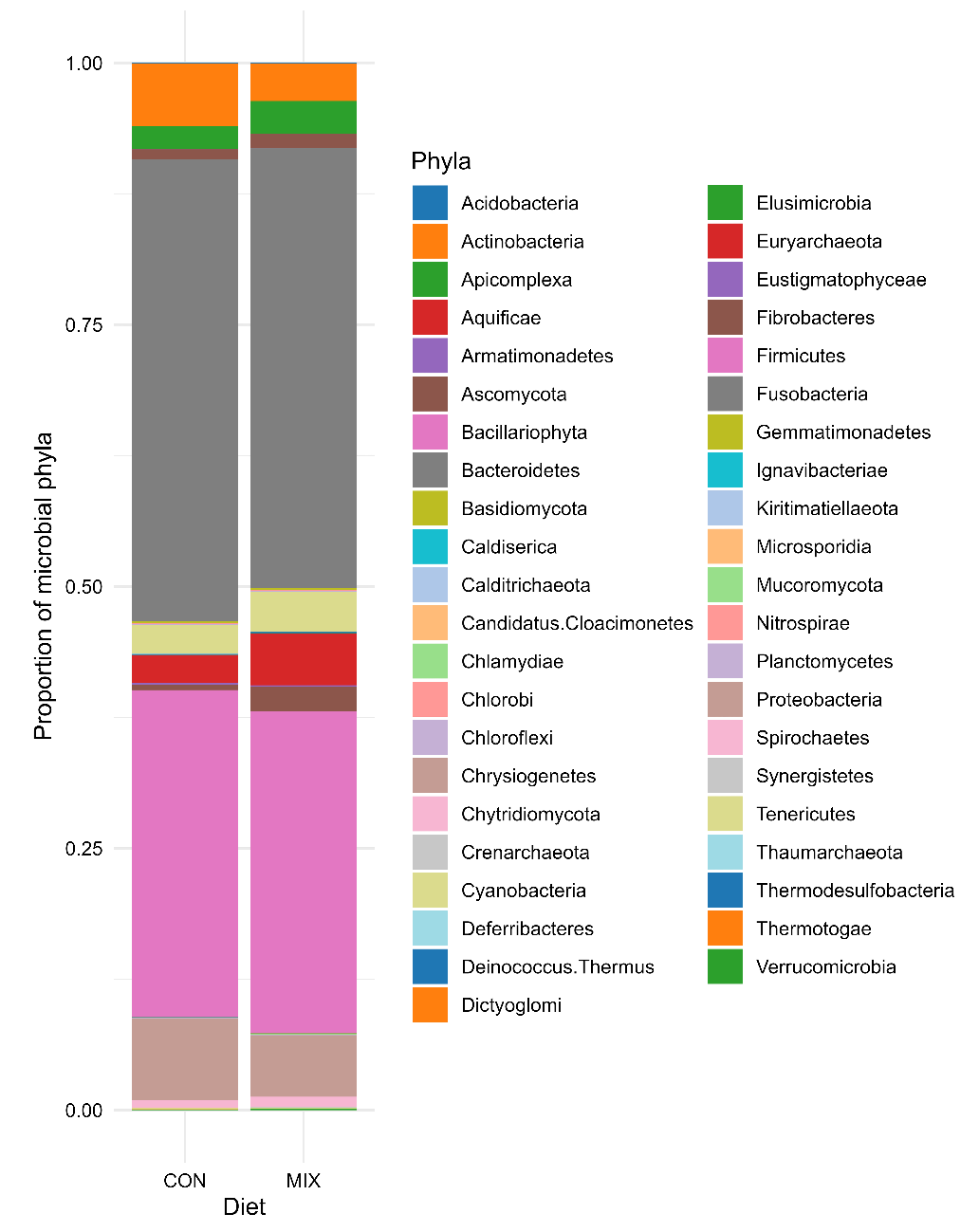


**Fig. S1** Relative abundance of microbial phyla across two diet types (*n* = 142). Stacked bar plots show the relative abundance (as a proportion from 0 to 1) of the 43 identified microbial phyla in animals fed two different diets: CON (concentrate-based, *n* = 72) and MIX (mixed-based, *n* = 70). Only phyla with a mean relative abundance greater than 1% are shown individually; phyla with lower abundances were grouped into the category “Others”.
